# Effect of intra-articular administration of superparamagnetic iron oxide nanoparticles (SPIONs) for MRI assessment of the cartilage barrier in a large animal model

**DOI:** 10.1371/journal.pone.0190216

**Published:** 2017-12-29

**Authors:** Raphael Labens, Carola Daniel, Sarah Hall, Xin-Rui Xia, Tobias Schwarz

**Affiliations:** 1 School of Animal and Veterinary Sciences, Faculty of Science, Charles Sturt University, Wagga Wagga, New South Wales, Australia; 2 The Roslin Institute, Easter Bush Campus, The University of Edinburgh, Midlothian, United Kingdom; 3 Animal & Veterinary Sciences, Scotland’s Rural College, Easter Bush Campus, Midlothian, United Kingdom; 4 Department of Biological Sciences, North Carolina State University, Raleigh, North Carolina, United States of America; 5 Royal (Dick) School of Veterinary Studies, Easter Bush Campus, The University of Edinburgh, Midlothian, United Kingdom; Argonne National Laboratory, UNITED STATES

## Abstract

Early diagnosis of cartilage disease at a time when changes are limited to depletion of extracellular matrix components represents an important diagnostic target to reduce patient morbidity. This report is to present proof of concept for nanoparticle dependent cartilage barrier imaging in a large animal model including the use of clinical magnetic resonance imaging (MRI). Conditioned (following matrix depletion) and unconditioned porcine metacarpophalangeal cartilage was evaluated on the basis of fluorophore conjugated 30 nm and 80 nm spherical gold nanoparticle permeation and multiphoton laser scanning and bright field microscopy after autometallographic particle enhancement. Consequently, conditioned and unconditioned joints underwent MRI pre- and post-injection with 12 nm superparamagnetic iron oxide nanoparticles (SPIONs) to evaluate particle permeation in the context of matrix depletion and use of a clinical 1.5 Tesla MRI scanner. To gauge the potential pro-inflammatory effect of intra-articular nanoparticle delivery co-cultures of equine synovium and cartilage tissue were exposed to an escalating dose of SPIONs and IL-6, IL-10, IFN-γ and PGE_2_ were assessed in culture media. The chemotactic potential of growth media samples was subsequently assessed in transwell migration assays on isolated equine neutrophils. Results demonstrate an increase in MRI signal following conditioning of porcine joints which suggests that nanoparticle dependent compositional cartilage imaging is feasible. Tissue culture and neutrophil migration assays highlight a dose dependent inflammatory response following SPION exposure which at the imaging dose investigated was not different from controls. The preliminary safety and imaging data support the continued investigation of nanoparticle dependent compositional cartilage imaging. To our knowledge, this is the first report in using SPIONs as intra-articular MRI contrast agent for studying cartilage barrier function, which could potentially lead to a new diagnostic technique for early detection of cartilage disease.

## Introduction

Physical and functional integrity of hyaline cartilage is paramount in the maintenance of joint health. When disturbances occur, chronic disease may ensue due to the tissue’s significant involvement in organ specific inflammatory pathways and its limited capacity for repair. The result, degenerative joint disease or osteoarthritis is associated with significant patient morbidities both in people and animals alike [1, 2]. Therefore, timely detection of ultrastructural cartilage change prior to the establishment of chronic disease and obvious imaging abnormalities represents a diagnostic target of tremendous significance.

Composed of chondrocytes and extracellular matrix (ECM), collagen and mostly glycosaminoglycans (GAGs), ultrastructural cartilage change is characterized by progressive ECM depletion. Consequently, due to fixed charge density changes of GAGs and a reduction in steric exclusion an increase in cartilage permeability is observed [3–5]. This principle is being exploited by compositional contrast enhanced magnetic resonance (MRI) and computed tomographic (CT) imaging techniques thereby allowing indirect determination of GAG content on the basis of contrast solute diffusion [6–8]. Similarly, nanoparticle enhanced μCT-imaging of the cartilage barrier function dependent on the particles’ surface charge has been reported [9]. However, reports of ultrastructural, compositional imaging based on intra-articular nanoparticle dependent steric exclusion could not be found at the time of writing.

Superparamagnetic iron oxide nanoparticles (SPIONs), allow particle enhanced MRI due to the particles’ magnetization potential and consequent strong imaging signal. In the context of joints, reports have been limited to methods of cell labelling and tracking [10–12] and the contrast enhancement of synovial tissues via phagocytosis of circulating SPIONs by reticuloendothelial cells [13, 14]. In the specific context of SPION enhanced MRI of articular cartilage a significant correlation between T2 signal and GAG content has been documented following intravenous contrast administration and under the reported imaging conditions [15].

Similar to that of other nanoparticles, toxicity of iron oxide nanoparticles is not solely determined by the dose administered but by a plethora of other characteristics including particle shape, size, surface chemistry or the protein-corona formation [16]. More recently, the pro-inflammatory potential of intra-articular SPION administration in mice has illustrated a short lived pro-inflammatory effect [17].

Seeking proof of concept and safety data in a large animal model, study objectives were to determine an appropriate SPION concentration for intra-articular particle enhanced MRI, to further evaluate the pro-inflammatory potential of SPION solutions on articular tissues and to demonstrate increased nanoparticle permeation and hence altered imaging signal (MR and multiphoton laser scanning microscopic imaging) in articular cartilage following ECM depletion.

## Materials and methods

Use of animal tissues and blood were reviewed and approved by the School of Veterinary Medicine Ethical Review Committee at The University of Edinburgh (Reference numbers: 31–14, 07–15, 22–15). Use of surplus biological material from hospitalized patients was permitted by owner consent.

### SPION synthesis and characterization

SPIONs were synthesized using a previously described citrate-capping method with minor modifications [18, 19]. A reaction mixture composed of 1.622 g of FeCl_3_ 6H_2_O and 0.596 g of FeCl_2_ 4H_2_O was dissolved in 40 mL pure water under argon protection. The pure water was prepared from deionized water by using a Dracor High Purity Water System (Durham, NC, USA). A dark suspension of Fe_3_O_4_ was obtained by addition of 5.00 mL concentrated aqueous ammonia (28%) into the solution under mechanical stirring at 360 rpm for 10 min. The citrate-capped Fe_3_O_4_ was obtained by addition of 4.40 g sodium citrate into the suspension and heated to 90°C under argon protection and constant stirring at 360 rpm for 30 min. After cooling to room temperature, the suspension was washed twice with ethanol (200 proof) and centrifugation at 3500g to remove excess free citrate. The suspension was washed three times with pure water and the aid of magnetic precipitation using a neodymium magnet. Finally, the precipitate was dispersed in pure water at 1 mg/mL (stock solution) for further application. The 3rd wash solution was retained as a vehicle control in subsequent *in vitro* experiments. Prior to administration, particle and vehicle control solutions were passed through a 0.2 μm filter and exposed to UV light for a duration of 12 hours. To recalibrate expected nanoparticle doses post filtration, ICP-OES analysis of 8- serial dilutions of the stock solution (expected Fe concentrations of 1000, 500, 250, 100, 50, 25, 5 and 1 μg/mL) was performed using a Perkin Elmer Model 8000 Dual View (Waltham, MA, USA). Observed Fe concentrations were compared to expected levels and the mean percent discrepancy calculated. A dosing sample obtained in later *in vitro* experiments and assessed for observed Fe concentrations validated the proposed concentration adjustment. Doses reported hereafter represent adjusted concentrations.

#### Transmission electron microscopy (TEM)

The SPION stock solution was diluted 20 times for TEM analysis. A drop of the diluted solution was added to a copper grid, the excess blotted with a filter paper and the grid allowed to dry overnight in a fume hood. The TEM image of SPIONs was acquired by using a JEOL 2010F High Resolution TEM (Peabody, MA, USA) at operation voltage of 200kV. The particle size analysis of the TEM image was conducted by measuring the diameter of 172 particles using NIH ImageJ.

#### Dynamic light scattering (DLS)

The hydrodynamic diameter of SPIONs was measured by using Zetasizer® Nano ZS equipment with a He/Ne 633 nm laser (Malvern Instruments, Malvern, UK). A serial dilution of the stock solution with water to different expected concentrations of SPIONs (2.2, 11.2, 22.4, 44.8 and 56 μg/mL) was measured at ambient temperature (22°C) using a microcuvette. Three replicated measurements were made for each of the sample solutions with a scan number automatically determined by the instrument. The reported data were the average results of the three replicated measurements made for each sample.

### Concentration dependent MRI signal of SPION solutions

Circular wells of approximately 0.6 cm diameter and 1 cm depth were formed in 1% agarose gels to simulate the in vivo cartilage/joint fluid interface on MRI examinations. Wells received 1 mL of an escalating nanoparticle dose solution (2.2, 11.2, 22.4, 44.8 and 56 μg/mL) while control wells were filled with 1 mL of water. Transverse T1, T2 and T2* weighted (W) frontal and dorsal sequences were obtained using a 1.5 Tesla field strength MRI scanner (Philips Achieva™, Philips Medical Systems, Reigate, UK) and the minimum nanoparticle concentration resulting in noticeable change in MRI signal compared to that of water and agarose gel was determined. The details of the MRI sequences performed are listed in Table 1.

**Table 1:** Technical parameters of the performed MRI scans.

MRI Parameters	T1	T2	T2*
Slice Width	2mm	2mm	2mm
Echo Time (TE)	12ms	125ms	15ms
Repetition Time (TR)	647ms	2267ms	276ms

### Barrier function of unconditioned and conditioned porcine cartilage to fluorescently labelled AuNPs assessed by multiphoton laser scanning microscopy

Distal limbs (trotters) were obtained from adult sows at a local abattoir (name available upon request) and sterilely dissected to remove all periarticular soft tissues and to preserve the 2^nd^ and 5^th^ metacarpo(tarso)phalangeal joints. To evaluate the barrier function of unconditioned cartilage, joints were injected with 100 μl of a fluorescently labelled (AlexaFluor® 532, ThermoFisher;, Waltham, USA), PEGylated, 30 and 80 nm, spherical AuNP solution [Nanopartz Inc, Loveland, USA; C16A1-30(80)-532-PEG-50; 4.5 (2.6) mg in 1mL] in 0.6 mL of Ultrapure™ water (ThermoFisher; 10977023). To evaluate the barrier function of conditioned cartilage i.e. when ECM components have been depleted, whole organs were cultured for two weeks prior to intra-articular nanoparticle administration. Joints were submerged in high glucose Dulbecco's Modified Eagle Medium (DMEM, Gibco™, ThermoFisher; 11965) with 100 U/μg/mL of penicillin-streptomycin (Gibco™, ThermoFisher; 15070063) and 10% v/v fetal bovine serum added (Sigma; St. Louis, USA; F0804) and maintained in a 5% CO_2_ and water saturated atmosphere at 37°C. A complete growth media exchange was performed twice per week. Post intra-articular nanoparticle administration all joints were manually cycled for 100 times, disarticulated and the articular surface of the first phalanx mounted in a petri dish, submerged in PBS. Multiphoton laser scanning microscopy (MP-LSM; MP7, Carl Zeiss Ltd, Cambridge, UK—max. laser power 3570 mW) of an area abaxial to the sagittal groove of the first phalanx was performed using the following settings: laser wavelength 840 nm; laser line attenuator transmission 10%; frame size X1024 Y1024; speed 7; pixel dwell 1.58 μsec; scan time 7.75 sec; averaging number 2; bit depth 8 bit; slices 40; mean interval 7.01 μm (SEM 0.83 μm); mean penetration depth 262.89 μm (SEM 30.04 μm); blue channel gain 548–592; green channel gain 683. Contemporaneous 3^rd^ or 4^th^ metacarpo(tarso)phalangeal joints were conditioned in the same way but not injected with nanoparticles and prepared for live/dead cell staining and MP-LSM. For this, sections of the articular surface of the first phalanx containing cartilage and underlying subchondral bone were obtained and stained with 5-chloromethylfluorescein diacetate (Cell Tracker™ Green CMFDA, ThermoFisher; C7025; live cells = green) and propidium iodide (Molecular Probes™, ThermoFisher; P3566; dead cells = red). 75 μl of a 1 mg/mL CMFDA/DMSO (Sigma; P1037) and PI solution were added to 150 mL of high glucose DMEM containing 100 U/mg/mL of penicillin-streptomycin. The solution was aliquoted in suitable containers and samples submerged for 75 min at room temperature. Subsequently specimens were fixed in 4% formaldehyde in 0.9% v/v NaCl for 24 hours at 4°C. Samples were then transferred into PBS and mounted in petri dishes for MP-LSM adopting previously mentioned settings except for: laser line attenuator transmission (7.5%;) red channel (762); blue channel (636) and green channel (666) gain. Mean intervals were 4.96 μm (SEM 0.3 μm) and the mean penetration depth 193.46 μm (SEM 11.52 μm). On MP-LSM fluorescently labelled nanoparticles were represented as green signal, while blue signal was associated with the second harmonic generation principle, indicative of collagen content [20, 21]. Unconditioned and conditioned control joints were processed in an identical way but injected with 0.6 mL of an AlexaFluor® 532 water solution (1 mg/mL) to compare imaging findings. Post imaging, joint samples were fixed in 4% formaldehyde in 0.9% v/v NaCl for one week and then decalcified in neutral ethylenediaminetetracetic acid (EDTA) solution for further processing. Tissue sections subsequently underwent H&E staining, autometallographic enhancement of permeating AuNPs for 25 minutes (Goldenhance™-LM/Blot, Nanoprobes, Yaphank, USA) and Alcian blue staining to evaluate GAG loss in conditioned vs. unconditioned joints. Analysis of histologic stains and fluorescent nanoparticle associated MP-LSM signal was performed descriptively. Determination of live/dead cell ratios in cartilage samples of contemporaneously conditioned joints was performed using Imaris® (Bitplane, Zürich, Switzerland) and the following standard settings for automated 3D processing: point styles—spheres; radius scale—1.0; render quality—50%; estimated diameter dead cells (= red channel—6 μm; estimated diameter live cells (= green channel) - 8 μm. Automated selections were reviewed in each image and manually corrected if required to reflect appropriate determination of cells.

### MRI assessment of barrier function of unconditioned and conditioned porcine cartilage to SPIONs

Porcine trotters were retrieved and processed in the same way as described above to provide conditioned and unconditioned isolated joints. Submersed in a water bath, joints were imaged in a sagittal plane obtaining T1, T2 and T2* weighted MR image sequences prior to and again after SPION administration at a concentration identified earlier and manual cycling (100 times). MR images were imported into an open-source DICOM medical image viewer (Horos™) and the brightness (WL) and contrast (WW) normalized across imaging studies (420/821). The first sagittal slice abaxial to the condylar sagittal ridge, also showing the palmar/plantar proximal sesamoid bone best, was selected and the image exported as a TIFF file for further processing in ImageJ 1.50g (National Institutes of Health). Once imported, the image was calibrated, the grey scale intensity of the metacarpal/tarsal cartilage profiled along a line drawn perpendicular to the distal metacarpal/tarsal physis and the data represented as a profile plot (x axis = distance in mm; y = intensity scale). Subsequently, the “peak” grey scale intensity point was identified just prior to the profile plot dropping off at the cartilage-joint cavity interface. From this, the “half-peak” grey scale intensity point was determined on the plot and the distance to the peak point recorded (in mm). The difference in this value between pre- and post-nanoparticle administration was used in statistical comparisons between conditioned and unconditioned joints and further expressed as a percentage of the pre-injection value.

### Pro-inflammatory and chemotactic response of equine articular tissues exposed to SPION solutions

To assess the nanoparticles’ potential effect after intra-articular administration in the context of local inflammation and cell recruitment, growth media from cartilage and synovial tissue co-cultures exposed to SPION was assessed for relevant cytokine content and utilized in transwell migration assays of isolated equine neutrophils.

#### Tissue cultures

In horses euthanized by intravenous barbiturate overdose for unrelated research at the University of Glasgow (University research ethics committee approval– 25a/13) sheets of cartilage were shaven off the subchondral bone plate of the femoral trochlear ridges and the patella. Synovial membrane samples were obtained by resecting the dorsal synovial fold in metacarpo(tarso)phalangeal joints. All samples were obtained in an aseptic manor, and stored in Geys balanced salt solution (GBSS, Sigma; G9779). Using a 6 mm skin biopsy punch, discs of cartilage were prepared from larger cartilage sheets. Harvested synovial folds were trimmed into 1 cm strips and all tissue samples washed in GBSS before plating. Wells in tissue culture plates were stocked with four randomly selected cartilage discs and one strip of synovial fold each and 9 mL of Hams F-12 (Life technologies, Carlsbad, USA; 21765) supplemented with 2 mg of ceftiofur (Zoetis, London, UK; 42058/4066) were added. Treatment groups were dosed with either 22.4 μg (Group L), 112 μg (Group M) or 560 μg (Group H) of SPIONs in 1 mL of vehicle solution or with 1 mL of the vehicle control solution (Group CW). Separate wells controlling for the effects of growth media (only media and no tissues in wells) and treatment (tissues but no particles or vehicle solution added to wells; Group C) were dosed with a sham treatment (1 mL of ultrapure water; Cayman Chemical, Ann Arbor, USA; 400000) to establish the same growth media volume across all experimental groups. Culture plates were incubated in a 5% CO_2_ and water saturated atmosphere at 37°C and after 12 hours a complete growth media exchange was performed. Wells were refilled with 10 mL of Hams F-12 and 2 mg of ceftiofur and an additional 12-hour culture period was completed after which media samples were collected, flash frozen and stored at -80°C until further processing. Synovial and cartilage tissue samples were weighed and one randomly selected cartilage disc prepared for live/dead cell staining using MP-LSM. The same settings as described earlier for evaluation of fluorescently labelled AuNP were adopted except for the mean (SEM) green and red channel gain setting which were 686 (13) and 847.5 (9.1) respectively. The penetration depth and slice interval were variable depending on the individual specimen [Mean (SEM); 209.9 μm (17 μm) and 5.4 μm (0.4 μm)]. Automated 3-D image processing to determine live/dead cell ratios was accomplished as in previous analyses.

#### Neutrophil isolation and transwell migration assays

Neutrophils were isolated on the basis of density gradient centrifugation. Per animal 25 mL of equine whole blood was utilized which was collected during placement and maintenance of an indwelling catheter into the jugular vein to facilitate routine veterinary procedures and diagnostic tests. Placement of catheters was performed by veterinary professionals adhering to accepted standards for this procedure (i.e. use of local anaesthetic, appropriate animal restraint and sterile placement). In short, heparinized blood was aliquoted in 15 mL polypropylene tubes (Sarsted, AG & Co, Nümbrecht, Germany; 62.554.502) and left to settle for 45 minutes to generate leukocyte rich plasma. 10 mL of leukocyte rich plasma was then layered on top of 5 mL of Ficoll-Paque Plus™ (Sigma, GE; 17-1440-02) and centrifuged at 1800 rpm for 20 minutes. The supernatant was rejected and residual erythrocytes in the cell pellet were removed by hypotonic lysis for 60 seconds. On the basis of Wright’s Giemsa staining (Sigma; WG16) isolation performance was assessed and viability and cell numbers were determined using trypan blue dye exclusion (Sigma; T8154) and a manual haemocytometer count. Isolated neutrophils were subsequently re-suspended at 1 x 10^7^/mL of sterile HBSS (Life technologies; 14175) and incubated with calcein (Sigma; C1359) for 30 min at 2 μg/mL. Calcein stained cells were washed twice (two-time centrifugation at 1000 rpm for 8 minutes and resuspension in HBSS), and prepared to their final density at 2 x 10^6^ cells/mL of chemotaxis buffer containing 1 mM of calcium and magnesium and 5% FBS (Sigma; F0804) in HBSS^++^ (Life technologies; 14025). Transwell migration assays were performed using a 3 μm pore size, 96-well NeuroProbe Chemotx® plate system (Receptor technologies, Warwick, UK; 101–3). For this, each dosing position on top of the separating filter membrane received 4 x 10^4^ calcein stained cells in 20 μl of chemotaxis buffer. Cells were allowed to migrate for 1 hour at 37°C toward bottom wells containing 30 μl of either chemotaxis buffer; HBSS^++^ with chemoattractant (Leukotriene-B_4_, LTB_4_, Sigma; L0517) at 1 μg/mL, 0.1 μg/mL and 0.01 μg/mL, growth media from tissue co-cultures or HBSS^++^ with LTB_4_ vehicle (EtOH). Following incubation residual cells at the top position on the separating filter were scraped off, the filter gently rinsed with PBS, the plate centrifuged at 1000 rpm for 1 minute and read at 485 nm excitation and 538 nm emission (1420 multi-label counter Victor3, Perkin Elmer, Coventry, UK). Fluorescence in bottom wells loaded with the dosing cell solution (4 x 10^4^ cells in 20 μl) informed on maximum migration and facilitated relative interpretation of migrating cell proportions. Experiments were completed in triplicates within 4–5 hours of blood collection and run in cohorts i.e. samples were not distributed across plates to preserve the integrity of internal treatment controls. For further analyses fluorescence readings were averaged across repetitions.

#### Articular biomarker synthesis in tissue co-cultures

Levels of interleukin (IL)-6; IL-10 and interferon (IFN)-γ were determined utilizing a multiplex method described by members of the group elsewhere [22]. Previously frozen growth media samples were thawed on ice and diluted 2x in assay buffer, analysed in triplicates and data averaged across repetitions. Assays were validated by assessing repeatability (intra and inter-assay coefficient of variation; CV) and limits of detection (cytokine recovery and range). Values were reported when within the measurable concentration range, when associated quality controls showed a recovery rate between 70 and 130% and when the data points’ CV was < 15%. Prostaglandin E_2_ (PGE_2_) levels were assed using a competitive ELISA kit (Cayman Chemical; 514010) with samples assessed in triplicates after 2x, 10x, 25x or 30x dilution to fall within the recommended concentration range for optimal test performance. The plate’s absorbance was read at 405 nm (1420 multi-label counter Victor3, Perkin Elmer). Outliers that resulted in a CV > than 30% were excluded from the analysis.

### Statistical analyses

The combined weight of tissue samples per well was analyzed using one-way analysis of variance (ANOVA) to evaluate for differences in plate loading. Differences in pre- and post-injection distances between “peak” and “half-peak” intensity grey scale points were compared for conditioned and unconditioned joints using Kruskal-Wallis one-way ANOVA on ranks and the Dunn’s method for multiple comparisons. Total fluorescence of cells in transwell migration assays was compared between groups (Group L, M, H, C and CW) using one-way ANOVA with Group C set as the control group in multiple comparisons according to the Holm-Sidak method. Cytokine levels were compared between groups (Group L, M, H, C and CW) using Kruskal-Wallis one-way ANOVA on ranks and the Dunn’s method for multiple comparisons. Statistical tests were performed using SigmaPlot 12.3 (Systat Software Inc, San Jose, USA) and significance was set at P < 0.05 for all tests.

## Results

### SPION synthesis and characterization

**Fig 1:**
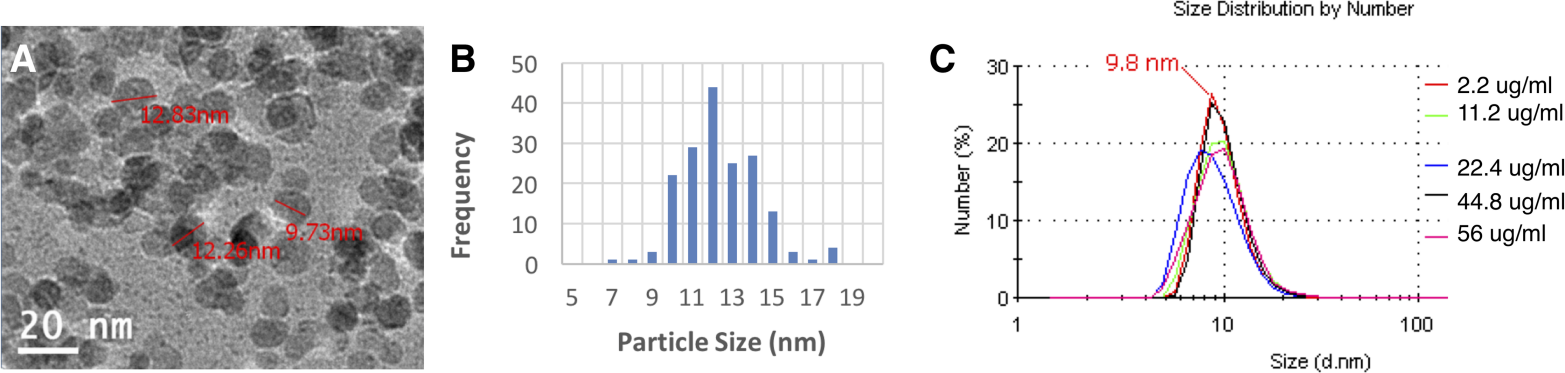
SPION characterization. A section of the TEM image of SPIONs after 20 times dilution in pure water (A). A histogram was obtained by particle size analysis of 172 particles on the TEM image using NIH ImageJ software (B). The size distribution of SPIONs in pure water at varying concentrations (2.2, 11.2, 22.4, 44.8, 56 μg/mL) was measured by using ZetaSizer® Nano ZS at 22°C (C).

Fig 1A shows a section of the TEM image of SPIONs after 20 times dilution in pure water. A histogram (Fig 1B) was obtained by particle size analysis of 172 particles on the TEM image using NIH ImageJ software. From the histogram the average particle size of SPIONs was estimated to be 12 nm. On the basis of DLS measurements the greatest proportion of SPIONs in the stock solution was 9.8 nm and this size varied little in the different test solutions (Fig 1C). ICP-OES analysis of elemental Fe in the dilution series and comparison with expected results illustrated that on average 44% of the nanoparticle mass was lost due to filtration. This loss is consistent with the observed Fe concentration in a dosing sample which expected to contain 40 μg of elemental Fe, measured 20.3 μg on ICP-OES analysis. Elemental Fe concentrations in the wash solution were found to be below detectable limits (Fe < 0.005 μg/mL).

### Concentration dependent MRI signal of SPION solutions

**Fig 2:**
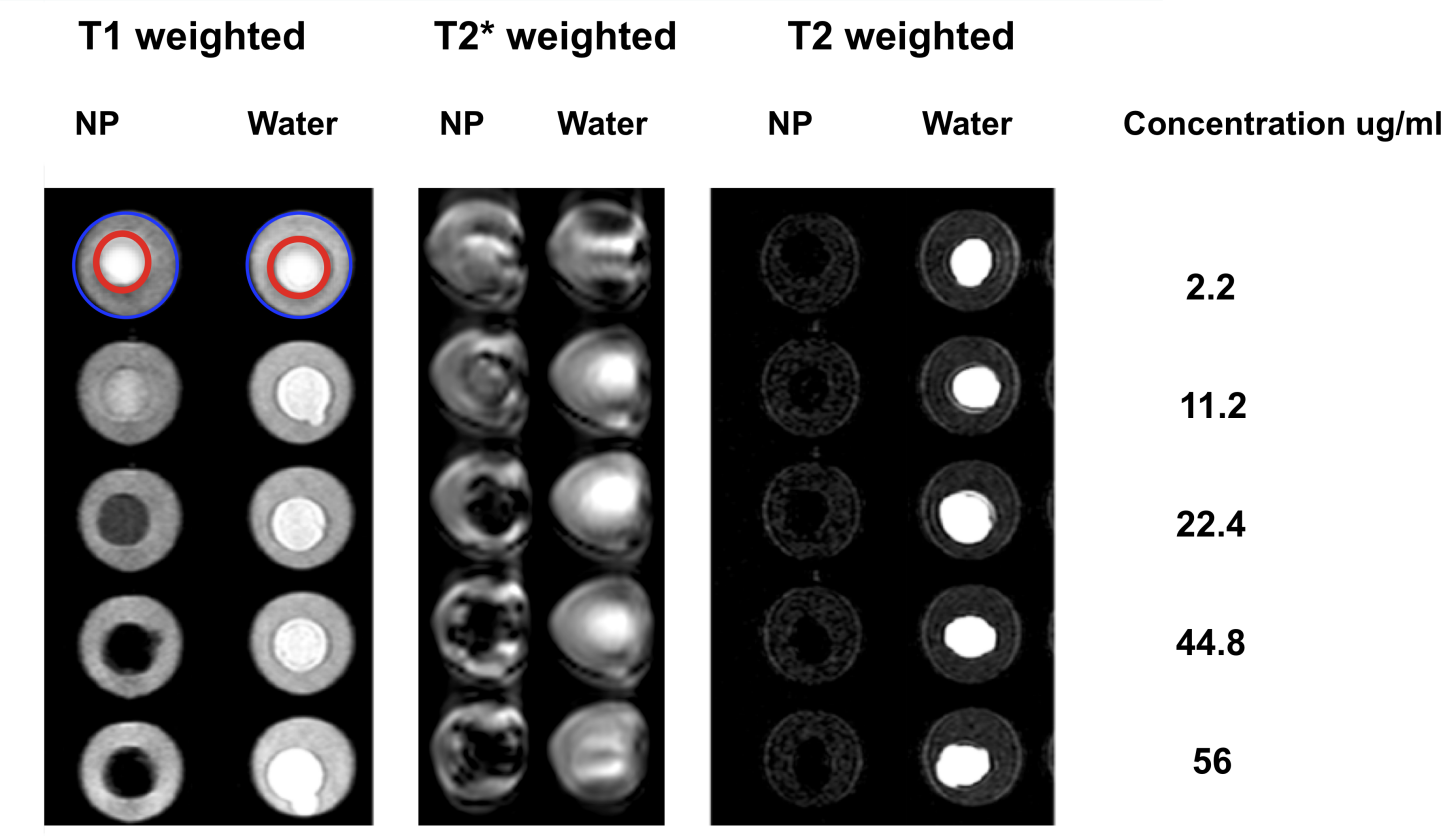
Concentration dependent T1, T2* and T2 weighted MRI signal of nanoparticle solutions and water. MRI signal associated with water or nanoparticle solution (located within the central, red circle) is contrasted against MRI signal of simulated cartilage (located between red and blue circles). Solutions at or above 22.4 μg/mL produced marked hypointense signal on T1, T2 and T2* weighted sequences in contrast to water or simulated cartilage.

MR imaging of cartilage/joint space phantoms (wells in 1% agarose gels filled with an escalating dose of SPIONs or water = area contained within red circles in Fig 2), determined that solutions at or above 22.4 μg/mL produce marked hypointense signal on T1, T2 and T2* weighted sequences in contrast to water or agarose gel (area contained between red and blue circles; Fig 2). Consequently, this minimum concentration to result in a marked change in imaging signal was evaluated further. Of the three sequences obtained, T2* weighted imaging resulted in the strongest signal alteration due to the susceptibility artefact created by the SPIONs.

### Barrier function of unconditioned and conditioned porcine cartilage to fluorescently labelled AuNPs assessed by multiphoton laser scanning microscopy

**Fig 3:**
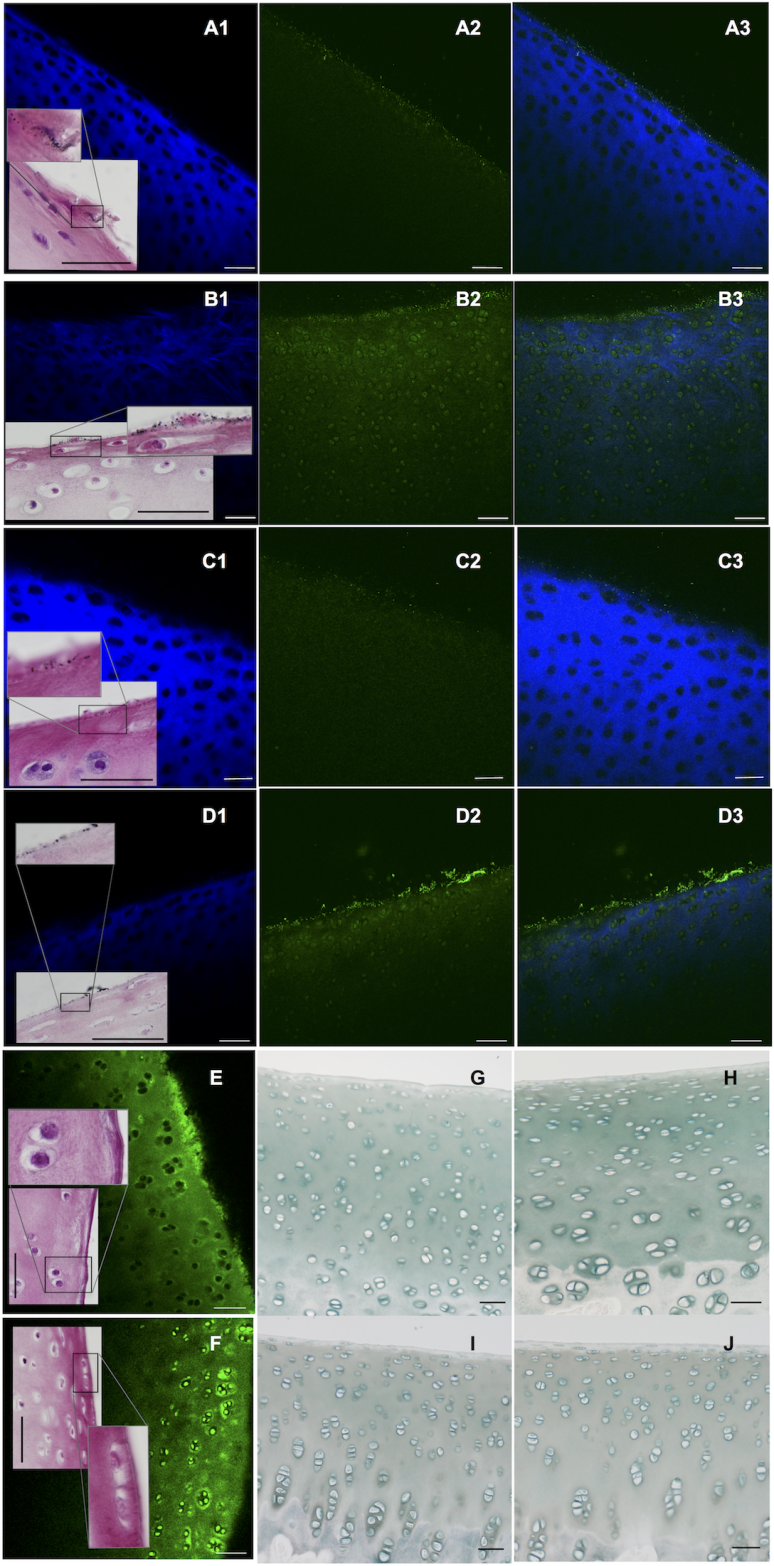
Multiphoton laser scanning and bright field microscopic images post autometallographic enhancement to assess nanoparticle permeation. Panels A-F show axial multiphoton laser scanning microscopic images of conditioned and unconditioned cartilage samples post fluorophore conjugated AuNP or free fluorophore exposure (green channel). Matched histologic images (H&E) post autometallographic particle enhancement provide an alternate method for AuNP-detection (dark signal; bar in panels = 50 μm). Panel A: Unconditioned cartilage exposed to 30 nm fluorophore conjugated nanoparticles. Panel B: Conditioned cartilage exposed to 30 nm fluorophore conjugated nanoparticles. Panel C: Unconditioned cartilage exposed to 80 nm fluorophore conjugated nanoparticles. Panel D: Conditioned cartilage exposed to 80 nm fluorophore conjugated nanoparticles. 1 = Second harmonic generation (SHG) signal in cartilage, representative of collagen content (blue channel). 2 = Nanoparticle bound fluorophore signal (green channel). 3 = Combined fluorophore and SHG signal. Panels E and F: Unconditioned (E) and conditioned (F) cartilage controls exposed to free fluorophore (green channel; matched histology included). Panel G and H: Alcian blue stains of unconditioned samples shown in A and C. Panel I and J: Alcian blue stains of conditioned samples shown in B and D. Superficial cartilage layers are permeable to 30 nm and to a lesser degree 80 nm particles. Conditioning induced GAG and collagen loss, seen here as reduced second harmonic generation imaging signal (blue channel) and loss of Alcian blue staining (Panel I and J). This led to deeper particle permeation and concurrent background staining/outlining of chondrocyte lacunae (Panel A2 vs B2; Panel C2 vs D2). Similarly post conditioning deeper permeation of 30 nm vs 80 nm fluorophore conjugated AuNPs was appreciated (Panel B2 vs D2). Controls exposed to free fluorophore showed marked ECM (Panels E and F) and intracellular fluorophore signal (Panel F), but absence of the very superficial “punctate” signal intensities as observed in Panels A2-D2.

MP-LSM of unconditioned and conditioned metacarpo(tarso)phalangeal joints injected with 30 nm (n = 4) and 80 nm fluorescently labelled spherical AuNPs (n = 3 unconditioned; n = 4 conditioned) showed that 30 nm and to a lesser extent 80 nm particle solutions breached the superficial cartilage barrier. Following conditioning and a loss of ECM as assessed by MP-LSM (collagen) and Alcian blue staining (GAGs), permeability and thereby particle associated imaging signal increased, resulting in peripheral and intracellular signal increase and an outline of chondrocyte lacunae (Fig 3). When compared with that of 30 nm particles, 80 nm particles were associated with less deep particle permeation in conditioned joints (Fig 3 Panel B2 vs. D2). Unconditioned and conditioned control joints (n = 4), injected with free fluorophore showed staining of the ECM and also intracellular fluorophore signal in conditioned joints resembling the image pattern with use of particle bound fluorophore in conditioned joints. In contrast to nanoparticle associated experiments, imaging post free fluorophore administration, did not show presence of superficial, “punctate” signal hyperintensities presumed to stem from larger AuNP agglomerates (Fig 3). To validate MP-LSM findings, autometallographic enhancement of permeating AuNPs was performed as an alternate imaging modality. On the basis of this modality conditioned cartilage showed greater particle permeation to 30 and 80 nm AuNPs, with unconditioned cartilage by enlarge reflecting 80 nm nanoparticle solutions.

**Fig 4:**
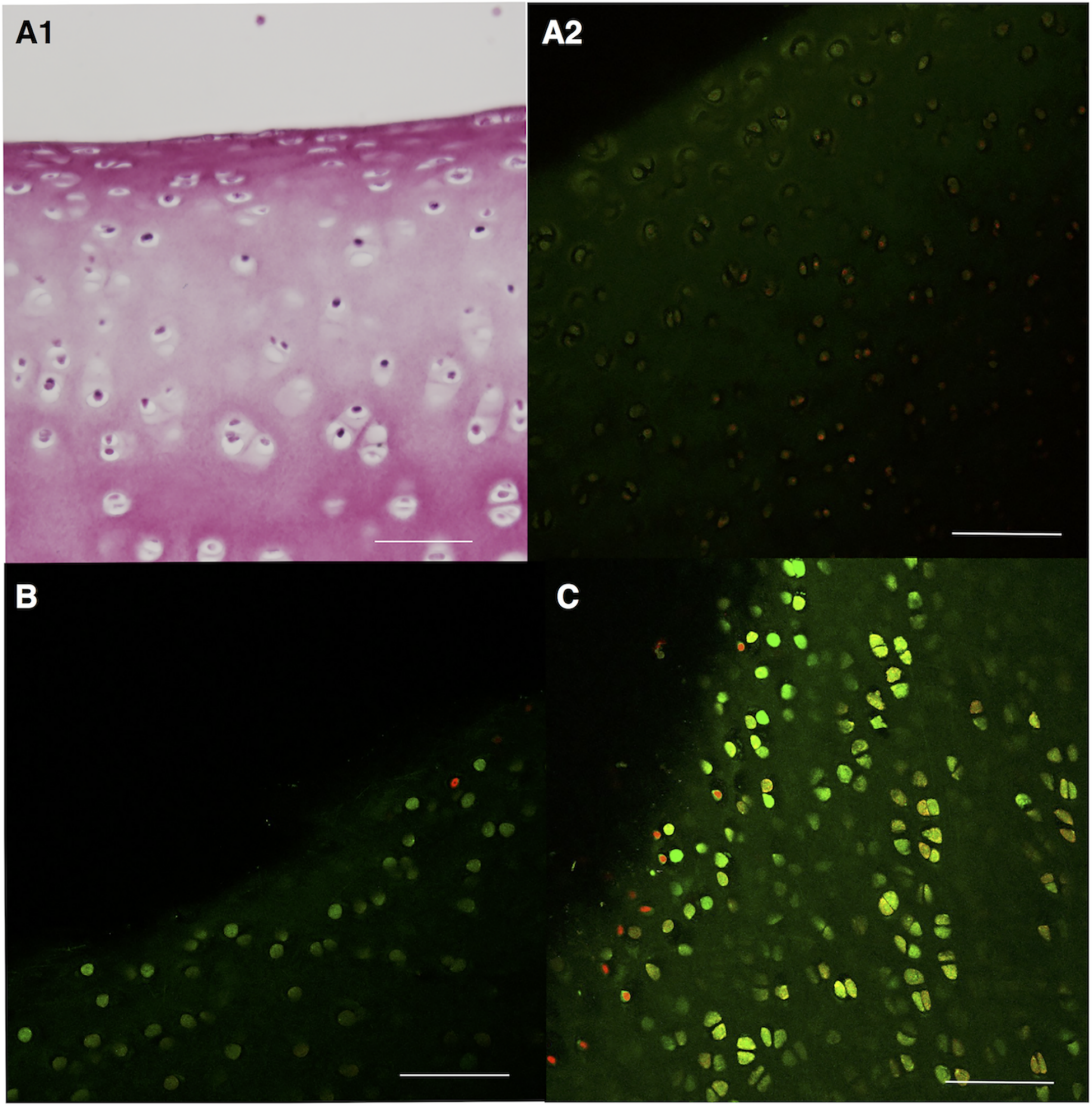
Cell viability after whole organ or tissue culture. Panel A1: H&E stain of a cartilage sample from a porcine conditioned joint also shown in Panel A2. Panel A2: Composite live/dead cell stain of cartilage from a conditioned porcine joint (after 2 weeks of whole organ culture). There is co-localisation of CMFDA (live; green) and propidium iodide (dead; red) signal but chondrocytes in superficial cartilage layers are predominantly CMFDA positive. Panel B: Live/dead cell staining of an equine cartilage sample exposed to low dose SPIONs (Group L). Panel C: Live/dead cell staining of an equine cartilage sample neither exposed to nanoparticles nor control vehicle solution (bar in panels = 80 μm).

Quantitative volumetric assessment of live/dead cell ratios in joints undergoing conditioning (whole organ culture) but not receiving intra-articular nanoparticles (n = 3) showed that on average 61.6% (SEM 7.5%) of cells were CMFDA positive. As shown in Fig 4 (Panel A2), live/dead cell staining of conditioned tissue samples showed some co-localisation of green/red fluorescence.

### MRI assessment of barrier function of unconditioned and conditioned porcine cartilage to SPIONs

**Fig 5:**
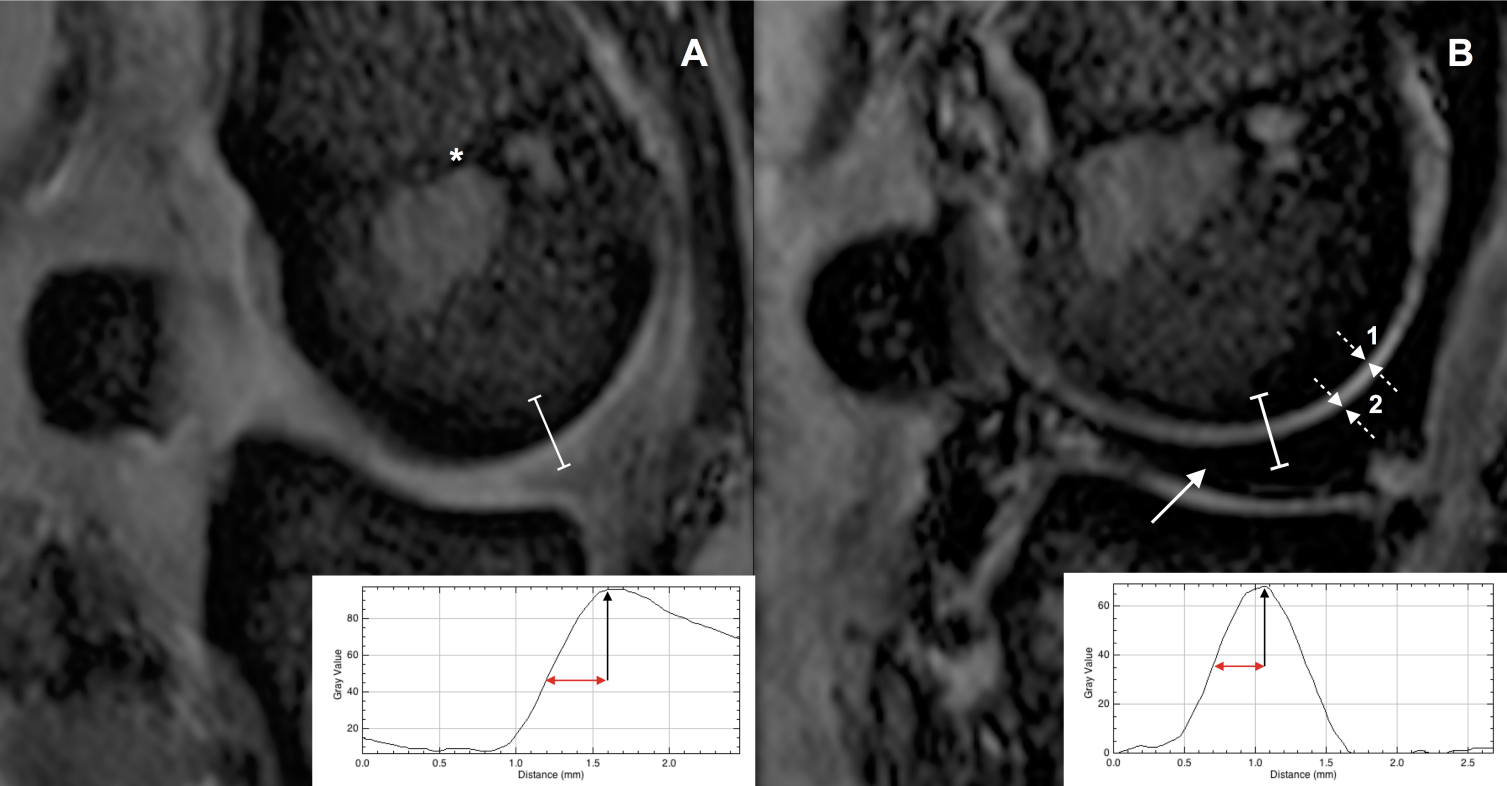
Particle dependent MRI signal and histologic findings. Sagittal T2*W MR images of a porcine metacarpo(tarso)phalangeal joint before (Panel A) and after (Panel B) SPION injection and joint cycling. Graph-inserts show the grey scale intensity plot profile measured along a line (white) through the midpoint of the condyle and at 90° to the physis (*). Bar = 50 μm. Following intra-articular injection resulting in hypointense filling of the joint (white arrow) and particle permeation, peak intensity grey scale levels of cartilage associated MRI signal (black arrow in graph insert) are reached at a shorter distance from the subchondral bone plate (number 1 in figure) suggestive of superficial signal change. This was assessed by measuring the distance between the “peak” and “half-peak” intensity grey scale point on the profile plot (red arrow in graph insert) and comparing this pre- and post nanoparticle administration. The cartilage surface is indicated by the number (2) in the figure.

High-field MRI was performed pre- and post intra-articular SPION administration in conditioned (n = 8) and unconditioned (n = 9) porcine joints and “peak” and “half-peak” grey scale intensity points and their distance in-between determined on the line profile plot (Fig 5, insert). When the difference (Value_pre_—Value_post particle administration_) was compared for conditioned and unconditioned joints a significant difference was observed (P = 0.001) in that in conditioned joints this distance was significantly shorter post- compared to pre-particle administration: The median percentage (first quartile; third quartile) of its original value being 59.57% (57.27%; 65.27%) in conditioned versus 86.99% (75.62%; 94.1%) in unconditioned joints. While in all conditioned joints peak intensity grey values were lower after SPION injection this was only the case in 5/9 unconditioned joints.

### Pro-inflammatory and chemotactic response of equine articular tissues exposed to SPION solutions

#### Tissue cultures

Tissues originated from five two-year old welsh ponies (two fillies and three geldings) giving rise to five growth media samples (n = 5) for each of the experimental Groups L, M, H and CW and 10 samples for Group C (n = 10). One-way ANOVA of tissue weight in culture wells did not show a significant difference in plate loading. The percentage of live chondrocytes in a randomly selected cartilage disc from a limited number of experimental groups (n = 2 for Group L; M; H; CW and n = 4 for Group C) showed on average (SEM) 86.5% (7.8%) viability in Group H; 77.1% (11.6%) in Group M; 86.9% (9.7%) in Group L; 66.7% (10.4%) in Group CW and 68.4% (14%) in Group C. Fig 4 illustrates the superficial cartilage area of two samples, one from Group C and one from Group L, after live/dead cell staining.

#### Neutrophil isolation and transwell migration assay

**Fig 6:**
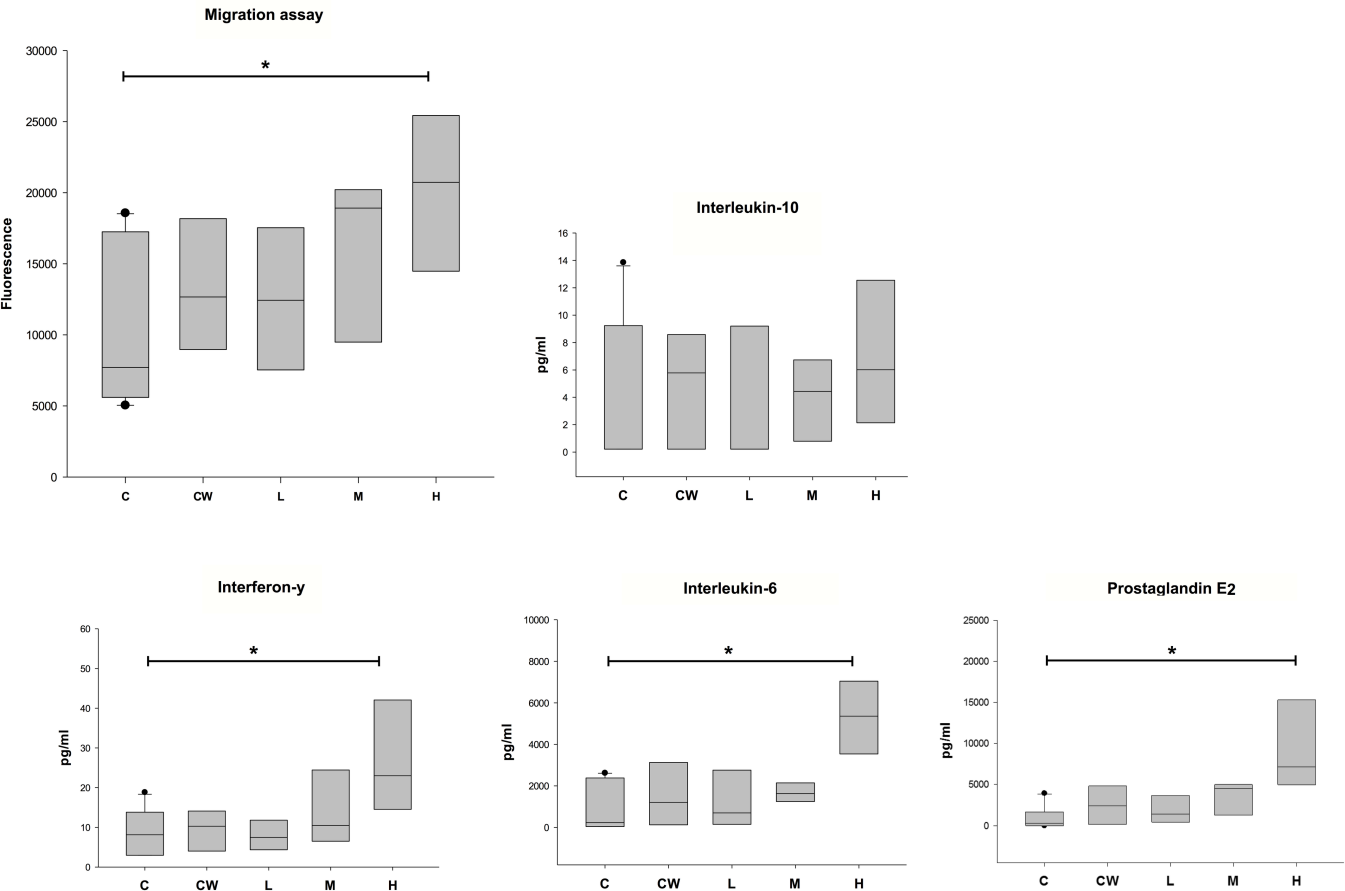
Chemotactic potential and cytokine content in culture media collected from cartilage and synovial tissue co-cultures post SPION exposure. C = Control tissues not exposed to nanoparticles (n = 10); CW = Vehicle control tissues exposed to nanoparticle vehicle (n = 5); L/M/H = Tissues exposed to nanoparticles at 2.5 (L), 12.4 (M) and 62.2 (H) μg/mL (n = 5). At the highest dose nanoparticle exposure is associated with greater neutrophil recruitment (P = 0.012), greater IL-6 (P = 0.011), Interferon-γ (P = 0.044) and PGE2 concentrations in growth media (P < 0.001).

Blood donors (n = 5) were of mixed breed, gender (four geldings and one mare) and age (average age 8.5 years). Isolated cells were largely neutrophils (>95% on Wright’s Giemsa staining) and their viability ≥ 99%. Results show that in respect to the tissue’s chemotactic response post SPION exposure, the administered dose had a significant effect (P = 0.041) with individual group means of total fluorescence (SEM) as follows: Group C—10076 (1768); Group CW—13390 (2350); Group L—12517 (2294); Group M—15666 (2623); Group H—20108 (2668). The trend that with an increase in nanoparticle dose, chemotaxis was also greater was significant for comparisons between Group H and Group C (P = 0.012; Fig 6). Average (SEM) fluorescence readings for migration controls were 11496 (1227) for LTB_4_ (1 μg/mL); 24046 (2669) for LTB_4_ (0.1 μg/mL); 26840 (2663) for LTB_4_ (0.01 μg/mL); 7132 (1012) for HBSS^++^; 19662 (2255) for 100% migration; 3958 (454) for growth media and 5335 (628) for LTB_4_ vehicle.

#### Articular biomarker synthesis in tissue co-cultures

When cytokine levels in tissue culture growth media were assessed (n = 5 for Group L, M, H and CW; n = 10 for Group C) a significant increase post SPION exposure was observed for IL-6 (P = 0.011); IFN-γ (P = 0.044) and PGE_2_ (P<0.001) but not for IL-10 (Fig 6). When present this effect was limited to comparisons between the response of control tissues and those exposed to the highest dose of SPIONs.

## Discussion

Diseased hyaline cartilage plays an important role in the development of osteoarthritis, which in its advanced stage is associated with readily detectable subchondral and peri-articular bone disease and cartilage erosions. Early recognition of joint disease at a time when cartilage dysfunction is limited to GAG depletion and failure of the collagen network leading to cartilage surface fibrillations represents a relevant diagnostic target in slowing disease progression [1]. Compositional cartilage imaging techniques aim to identify early disease on the basis of a charge dependent diffusion differential of contrast solutes into articular cartilage, either post intra-articular or intravenous administration. As GAGs contain negatively charged end-chains, contrast solutes are either attracted or repelled post administration of cationic or anionic contrast agents. With respect to compositional imaging of diseased cartilage this results in reduced (cationic) or increased imaging signal (anionic contrast agents) in areas of fixed charge density changes or reduced GAG content. Depending on the imaging modality and contrast used, these principles allow delayed gadolinium enhanced MR and contrast-enhanced computed tomographic imaging of cartilage composition [6–8, 23, 24]. While these modalities offer valid information, consideration of the partitioning equilibrium is required to accurately correlate cartilage associated imaging signal with GAG content. The concentration dependent diffusion coefficients governing the kinetics of this equilibrium are further affected by joint movement/ loading or the route of contrast administration (i.e. intravenous or intra-articular delivery), introducing complexities which may render diffusion rather than equilibrium based image analyses more practical [25–29]. As timing of contrast administration and imaging is critical for reliable compositional analyses, a contrast solution which post intra-articular administration is largely retained within the joint cavity would reduce clearance and loss of critical contrast concentrations. Furthermore, instead of detecting fixed charge density changes, use of a contrast agent which permeates cartilage based on steric exclusion and thereby primarily informs on the barrier function i.e. ECM depletion may reduce the effect of time on image acquisition and facilitate standardization and image interpretation. To this effect, a nanoparticle dependent contrast agent may satisfy the aforementioned requirements provided once injected into the articular environment, passage through the synovial membrane is limited and size of the nanoparticle reflects cartilage pore sizes encountered in disease.

So, extending the imaging principle of compositional cartilage imaging to the permeation of nanoparticles, the authors intended to demonstrate the feasibility of this approach for MRI. Initially, for MP-LSM assessment of cartilage barrier function, AuNP size selection was based on the assumption that 80 nm particle solutions would be largely reflected by healthy cartilage and synovial membrane while greater particle permeation would be expected with 30 nm particles or in diseased cartilage due to GAG and collagen loss and break-down of the barrier function. This assumption was supported by previous work which showed that nanoparticles of 20 nm to 38 nm in diameter were able to permeate into the ECM as opposed to larger particles which were by enlarge reflected by healthy cartilage (96 nm and 138 nm diameter) [30–32] and synovial membrane (50 nm) [33], a feature which may be of importance for maintenance of intra-articular contrast concentrations. Based on our initial findings and the increase in permeation of AuNPs of both sizes post conditioning, the general feasibility of the proposed imaging method was supported. For nanoparticle dependent MRI assessment of cartilage barrier function SPIONS were considerably smaller than AuNPs used in initial experiments, which one could argue would lead to unrestricted indiscriminate movement of particles into cartilage. However, in work by others, permeation of 15 nm micelles likewise increased with ECM depletion rendering the former assumption less plausible [32]. To summarize, given that post particle administration in conditioned joints, the peak intensity of cartilage associated MRI signal was reached at a closer distance to the subchondral bone and that it was generally lower than in unconditioned joints it can be concluded that nanoparticle permeation increased with GAG and collagen loss. However, unexpectedly 30 nm fluorophore conjugated AuNP permeation was only weak on MP-LSM and significant SPION associated MRI signal change was not detected in unconditioned metacarpophalangeal joints. As other reports have been in favour of permeation of similarly sized particles [30, 31], potential explanations for the perceived little or absent permeation in this work may range from particle agglomeration, protein corona formation [34] and insufficient spatial resolution of diagnostic imaging methods to the effects of negative surface charge and PEG-functionalization of AuNPs. With respect to the lack of significant MRI signal change following SPION administration it could also be argued that with use of smaller nanoparticles permeation was driven by ECM water exchange rather than steric particle exclusion. Collagen depletion has been shown to result in increased ECM water content due to unrestricted swelling of GAGs [3]. It is therefore conceivable that with this greater fluid exchange nanoparticles were consequently more readily “dragged” into the ECM resulting in the observed MRI signal change. In hind sight, representing a shortfall of this investigation, MRI post SPION administration had best been compared to findings post vehicle administration in the same joint. This would have allowed detection of increased fluid movement due to collagen depletion on the basis of cartilage associated T2* signal increase. However, this approach was not adopted due to the joint capsules leaking following repeat intra-articular injection of the same joint. Naturally, findings presented here only represent preliminary data and more work is required to correlate biochemical cartilage change such as GAG loss with nanoparticle dimensions and imaging signal to further this approach and validate the point of steric exclusion.

ECM depletion a hallmark of osteoarthritis was simulated by performing static whole organ cultures, a variation of a previously reported technique shown to preserve chondrocyte viability (98%) while leading to a 20% loss of GAGs within a comparable two-week culture period [35]. While in this study chondrocyte viability in superficial cartilage layers versus deeper portions was generally preserved as indicated by CMFDA dye uptake, others have recently shown that in injured cartilage sodium fluorescein staining pattern is also representative of dead cells or ethidium homodimer-1 uptake using MP-LSM [36]. As illustrated in Fig 4 (A2) observations made under the reported circumstances did not suggest a similar relationship between CMFDA and propidium iodide dyes. While live/dead cell stain intensities of conditioned cartilage were generally low and some co-localisation of imaging signal was also appreciated, a different signal pattern was observed for the two specific dyes in particular in the context of tissue depth.

Nanoparticle induced cytotoxicity is determined by a multitude of particle characteristics [16, 37, 38] explaining why 13 nm AuNPs can be chondrotoxic, as opposed to 3 and 45 nm citrate stabilized AuNPs [39], and SPIONs of a similar size have not been associated with a similar effect [17]. The limited viability data obtained in this investigation, a shortfall which should be acknowledged and results interpreted with caution, suggests that a cytotoxic effect was not observed post SPION administration. As such it could be suggested that the observed increase in superficial particle permeation into ECM was not a function of cell death and consequently lesser permeation hindrance but a direct result of ECM loss. This depletion of ECM was convincingly appreciated both based on the collagen specific MP-LSM signal, a result of the second harmonic generation principle [20, 21] as well as on histology with Alcian blue staining. While static whole organ cultures resulted in the desired outcome i.e. ECM loss, this model is arguably not representative of joints with naturally occurring early disease. However, justified by the observed results, future refinement of particle characteristics is expected to allow an increase in diagnostic performance and thereby facilitate detection of less advanced cartilage dysfunction.

Autometallography is a well-accepted technique for enhancement of permeating AuNPs thereby allowing detection of nanoparticles by bright field microscopy [40]. While nanoparticles were easily detected in the synovial membrane in this (S1 Fig) and another study [33] particle associated imaging signal within cartilage was less obvious than on MP-LSM. This may have been due to histologic processing, in particular decalcifying steps however nanoparticle washout into decalcifying solutions was not assessed to validate this hypothesis. Conversely, cartilage being a more effective barrier than synovial tissue, permeating particle fractions may have been of much smaller diameter than the solution’s average particle size thereby not allowing autometallographic detection as clearly. MP-LSM a technique performed on fresh tissue without the need for prior tissue processing reduces the potential for particle loss, preserves superficial nanoparticle deposits as seen in Fig 3 (D2 and D3) and so provided a distinct advantage.

Exploring the potential for future in vivo intra-articular administration the authors sought to obtain safety data on the SPIONS pro-inflammatory potential i.e. the tissue’s PGE_2_ and cytokine release and chemotactic signalling post exposure in a large animal model. To this effect co-cultures of equine synovium and cartilage were performed in the presence of control and SPION solutions. Tissue culture media from these experiments were subsequently tested in migration assays to obtain an indirect measure of the particles’ pro-inflammatory potential which was validated by concurrent analysis of select biomolecules. Results from these experiments supported a dose dependent inflammatory response of articular tissues post SPION exposure as illustrated by significant increases in PGE_2_, IL-6 and neutrophil recruitment. This supports findings by others who have shown a short lived pro-inflammatory potential of SPIONS on articular tissues including an increase in IL-6 levels [17]. PGE_2_ and IL-6 play pivotal roles in acute and chronic joint inflammation and cell recruitment [41–45]. IFN-γ a cytokine typically classified as pro-inflammatory has also been associated with significant anti-inflammatory effects in models of early onset inflammatory joint disease [46]. In the context of this investigation and the absence of an IL-10 response, a cytokine with homeostatic and anti-inflammatory effects [47], observed IFN-γ increases are in the authors opinion indicative of the tissues’ response to inflammation rather than an inherent positive particle dependent effect.

Although not the focus of this paper but suggested by the results, evaluation of neutrophil recruitment may prove a useful surrogate measure of the inflammatory potential of nanoparticles and facilitate screening of particle candidates prior to costlier biomolecular analyses. This investigation further emphasized the need for meaningful control solutions when investigating the effect of nanoparticles. Although not significantly elevated, SPION wash solutions were also associated with an inflammatory response which may implicate vehicle-contamination as a contributing factor be it due to the presence of chemical residues or endotoxin. To offset this possibility in preceding pilot experiments adopting similar methodology Polymyxin B was also added to AuNP exposed tissue cultures to function as a LPS-scavenger. However, despite this addition, a significant effect of Polymyxin B was not noted (S2 Fig). Hence the observed neutrophil recruitment and biomolecular response was unlikely due to endotoxin contamination similar to findings by others who failed to observe a reduction in pro-inflammatory response after SPION administration in Toll-like receptor-4 deficient mice [17].

Although results suggest that 22.4 μg of SPIONs was an effective and safe dose under the short-term conditions investigated, comparisons were made with control groups that also expressed an inflammatory profile albeit a smaller one than in the presence of SPIONs. As this may also be explained by stimulatory effects induced by tissue harvest and handling, ultimately only in vivo applications of the proposed methodology will determine the true short and long term effect of intra-articular SPION exposure in a large animal model. Results obtained in mice have suggested that despite the observed initial pro-inflammatory response no long term effects on joint health (14 days) may be expected [17], however longer observational periods would be needed to come to this conclusion with certainty.

Again, it should be re-iterated that many relevant aspects of nanoparticle delivery are still to be explored to promote clinical applications requiring a truly multi-disciplinary effort. In respect to the presented findings most importantly further particle characterization in the context of magnetization and the associated thermal response is needed [48]. If exposed to temperatures greater than 50–55 ^o^C chondrocyte viability has been shown to be significantly reduced such that any clinical application of ferric nanoparticles in a magnetic field would have to result in less thermal response than that [49].

## Conclusion

Given the findings presented herein, the expected utility of nanoparticle dependent compositional cartilage imaging and the significance associated with early disease diagnosis, the authors feel that an ongoing effort to that end is justified. The authors do acknowledge the preliminary nature of the presented data, some methodologic weaknesses and that further research into nanoparticle behaviour in the articular environment is needed to fully evaluate the potential of this approach.

## Supporting information

S1 Fig Synovial membrane post nanoparticle exposure, H&E staining and autometallographic particle enhancement. 30 nm AuNP exposure in unconditioned (A) and conditioned joints (B); 80 nm AuNP exposure in unconditioned (C) and conditioned joints (D); Synovial membrane tissue sample from a joint not exposed to particles (E); Bar = 50 μm. 30 nm particles permeate to a greater degree into synovial membrane and this is not affected by conditioning (dark superficial signal = enhanced particles).
(TIFF)

S2 Fig Pilot migration experiments on the use of Polymyxin B. Recruitment of isolated porcine neutrophils in response to media from porcine articular tissue (n = 4) exposed for 12 and 24 hours to 5 nm and 50 nm AuNPs (+/- Polymyxin B), did not show a significant LPS scavenging effect. Bars indicate significant comparisons at the different time points.
(TIF)

S1 Table Study data in excel spread sheet. (XLSX)
