# Supplementary figures and images for: Effect of intra-articular administration of superparamagnetic iron oxide nanoparticles (SPIONs) for MRI assessment of the cartilage barrier in a large animal model
Source: PLoS One. 2017 Dec 29;12(12):e0190216. doi: 10.1371/journal.pone.0190216 (PMC5747449; doi:10.1371/journal.pone.0190216)

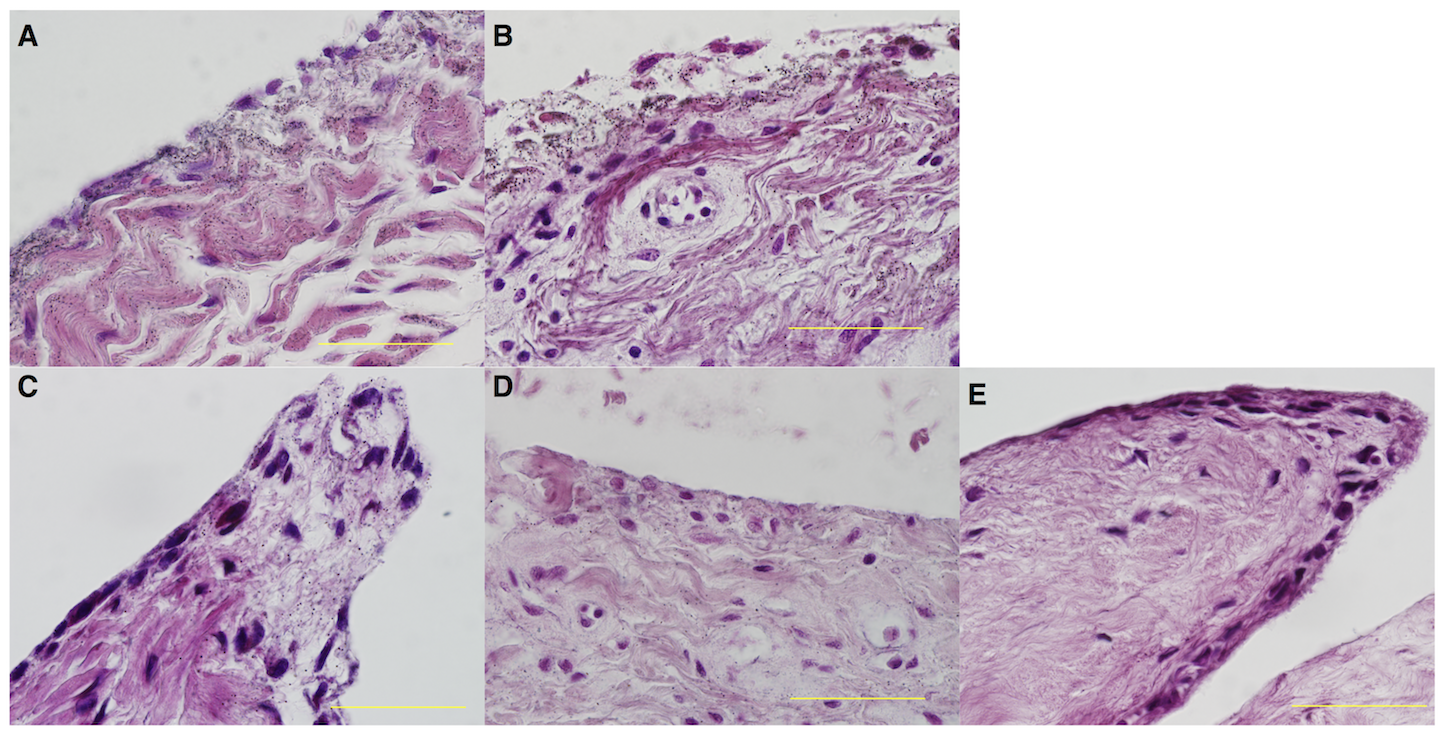

Supplement: S1 Fig — 30 nm AuNP exposure in unconditioned (A) and conditioned joints (B); 80 nm AuNP exposure in unconditioned (C) and conditioned joints (D); Synovial membrane tissue sample from a joint not exposed to particles (E); Bar = 50 μm. 30 nm particles permeate to a greater degree into synovial membrane and this is not affected by conditioning (dark superficial signal = enhanced particles). (TIFF) [file pone.0190216.s001.tiff]

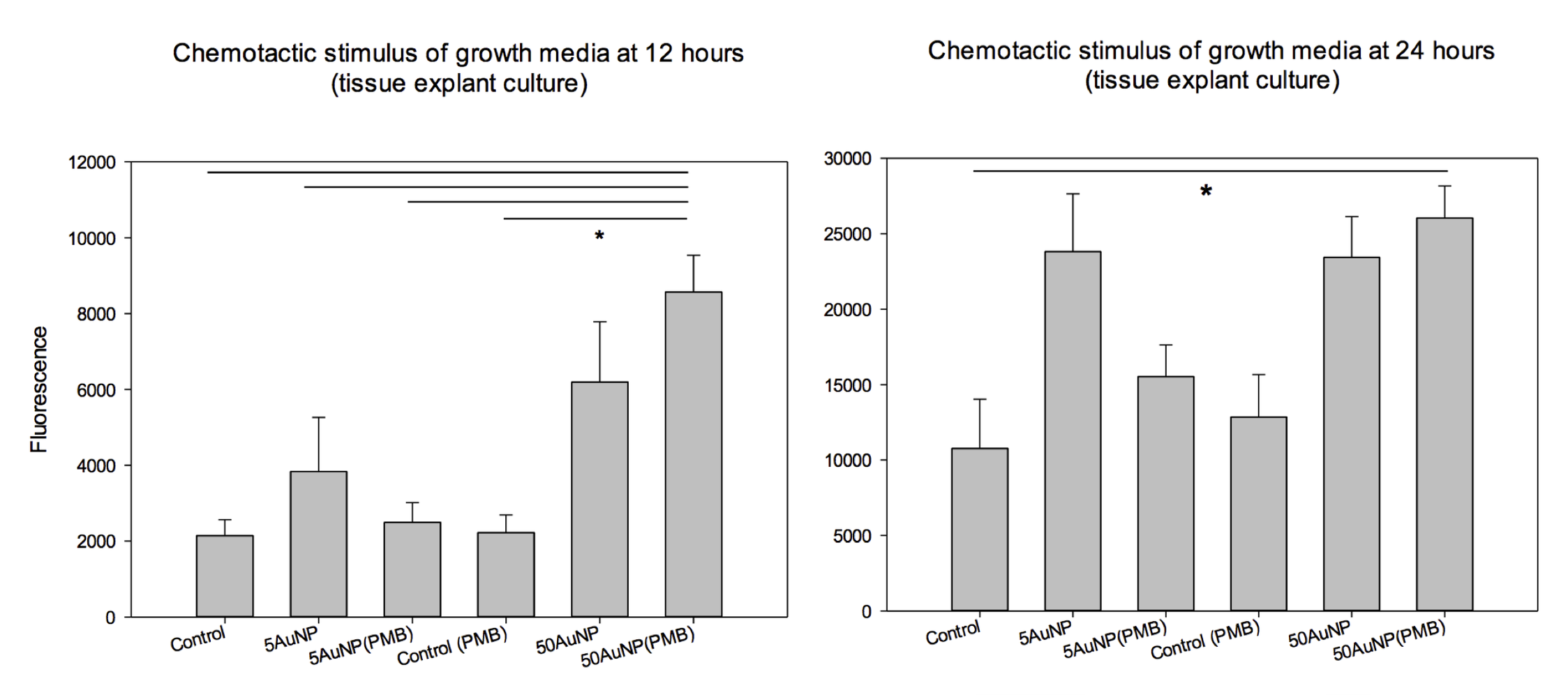

Supplement: S2 Fig — Recruitment of isolated porcine neutrophils in response to media from porcine articular tissue (n = 4) exposed for 12 and 24 hours to 5 nm and 50 nm AuNPs (+/- Polymyxin B), did not show a significant LPS scavenging effect. Bars indicate significant comparisons at the different time points. (TIF) [file pone.0190216.s002.tif]
